# A mini ROV-based method for recovering marine instruments at depth

**DOI:** 10.1371/journal.pone.0235321

**Published:** 2020-07-08

**Authors:** Joseph H. Tarnecki, William F. Patterson

**Affiliations:** Fisheries and Aquatic Sciences, University of Florida, Gainesville, Florida, United States of America; University of Waikato, NEW ZEALAND

## Abstract

Instruments are often deployed at depth for weeks to years for a variety of marine applications. In many cases, divers can be deployed to retrieve instruments, but divers are constrained by depth limitations and safety concerns. Acoustic release technology can also be employed but can add considerable expense and acoustic releases will at times fail. Here, we report a simple method that utilizes a commercially available mooring hook integrated with a mini remotely operated vehicle to attach lines to instruments deployed on the sea floor, which can then be winched to the surface. The mooring hook apparatus was tested in a pool setting and then used to retrieve acoustic telemetry receiver bases (50 kg) or fish traps (30–50 kg) from the northern Gulf of Mexico continental shelf at depths between 28 and 80 m. During 2013–2019, 539 retrievals (100% success rate) were made of receiver bases (n = 239) and traps (n = 300) on 30 sea days using this approach. This method could easily be applied to other types of instruments, or recovery and salvage of objects that are too deep for standard diving operations.

## Introduction

Marine research often requires deployment and retrieval of scientific instruments at depth, including on the seafloor. Instruments may require long soak times and rely on being tethered to surface floats for later retrieval [1], [2], [3], or require Self-Contained Underwater Breathing Apparatus (SCUBA) divers or other approaches to retrieve equipment [4]. Attaching instruments to surface buoys for long durations (months to years) can be problematic as boat traffic can displace equipment or sever mooring lines [5], [6], and theft of equipment can occur [1], [5], [6]. SCUBA can be a cost-effective means to conduct underwater research [7], [4], but there are associated risks for diver health due to myriad effects, such as barotrauma, decompression illness, pulmonary edema, or toxic effects of increased partial pressure of gases [7], [8]. Moreover, conventional SCUBA is limited to depths <40 m, and the need for technical divers diving on mixed gas or with closed circuit rebreathers at greater depths is more costly and incurs greater risk [7], [9].

An alternative to utilizing divers to recover instruments deployed at depth is to utilize acoustic release mechanisms to release instruments from benthic anchors, with attached buoys then bringing instruments to the surface [10]. However, this type of approach involves leaving weighted anchors on the seafloor that become marine debris. Acoustic release mechanisms are also costly, especially if numerous instruments are deployed at depth, such as deploying water quality instruments or building large-scale acoustic arrays over 10s to 100s of km^2^. Lastly, if acoustic mechanisms fail to release instruments, then a secondary approach is needed.

Here, we report an approach to retrieving instruments from depth that utilizes a mini remotely operated vehicle (ROV) to attach a line to an instrument at depth, after which the instrument is winched to the surface. In this approach, a mooring hook apparatus is integrated with the ROV. The hook is used to snatch lines attached to an instrument and then the ROV is flown away before the instrument is winched to the surface. We initially tested our design in a pool (S1 File.), then utilized our mooring hook apparatus practically in the field (S2, S3 and S4 Files) to recover a suite of instruments. We detail components of this system below, as well as describe examples of utilizing it to retrieve acoustic telemetry receiver bases and experimental fish traps from the seafloor in the northern Gulf of Mexico (nGOM).

## Materials and methods

### Remotely operated vehicle

**Fig 1:**
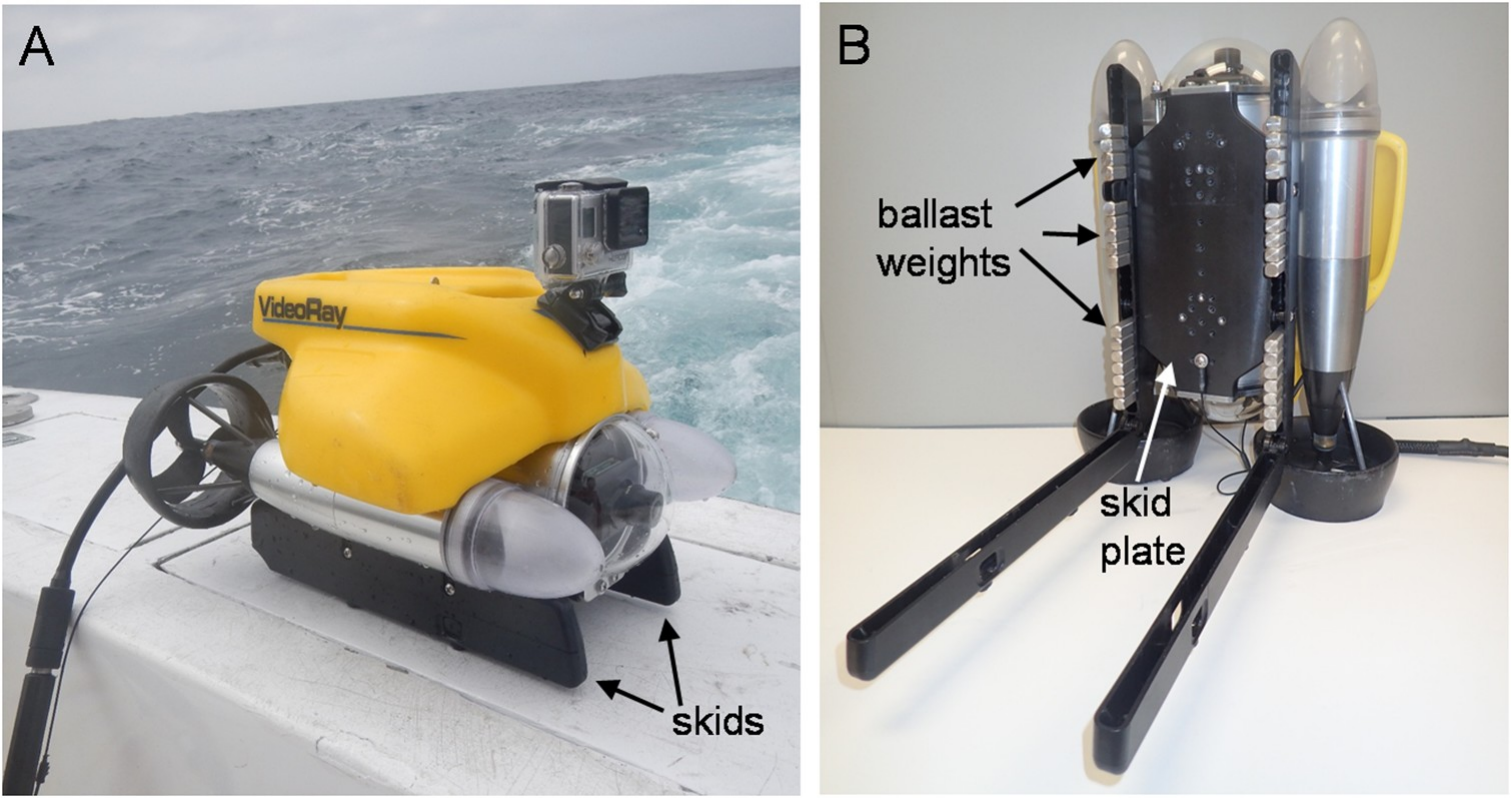
Remotely Operated Vehicle (ROV) ballast system. A) VideoRay Pro4 remotely operated vehicle (ROV) with skids labeled. B) Underside of the ROV with skids hinged open to reveal ballast weights. Also visible is the skid plate between the skids with tapped holes utilized for attaching ROV accessories.

The mooring hook apparatus (described below) was integrated with a VideoRay Pro4 mini-class ROV capable of diving to 305 m (Fig 1a; https://www.videoray.com/rovs/videoray-pro-4.html). The ROV’s dimensions are 37.5 cm x 28.9 cm x 22.3 cm, with a mass of 6.1 kg. It has two horizontal and one vertical thruster that enable the ROV to be flown in currents as great as 2.3 m‧s-^1^. The ROV is tethered to the surface and controlled by an integrated control box (ICB). The ICB contains a laptop PC that runs navigational software and is utilized to store digital video captured with the ROV’s forward facing camera. The ROV is designed with a plastic skid plate underneath that has an additional two ballast skids attached on which the ROV rests when not being flown. The skid plate has an array of threaded #8–32 screw holes to attach a variety of accessories (e.g., laser scaler, manipulator arm, imaging sonar, etc.) to be integrated with and controlled by the ROV. Flotation is provided by a hard syntactic foam shell. Ballast is provided by brass bars contained within the ballast skids. The ballast skids hinge open to reveal 25 slots that each can hold a 30-g bar (Fig 1B). The bars can be removed if mass is added to the ROV in the form of external camera housings or other accessories, and can be shifted to the front or back to maintain appropriate ballast.

### Mooring hook apparatus

**Fig 2:**
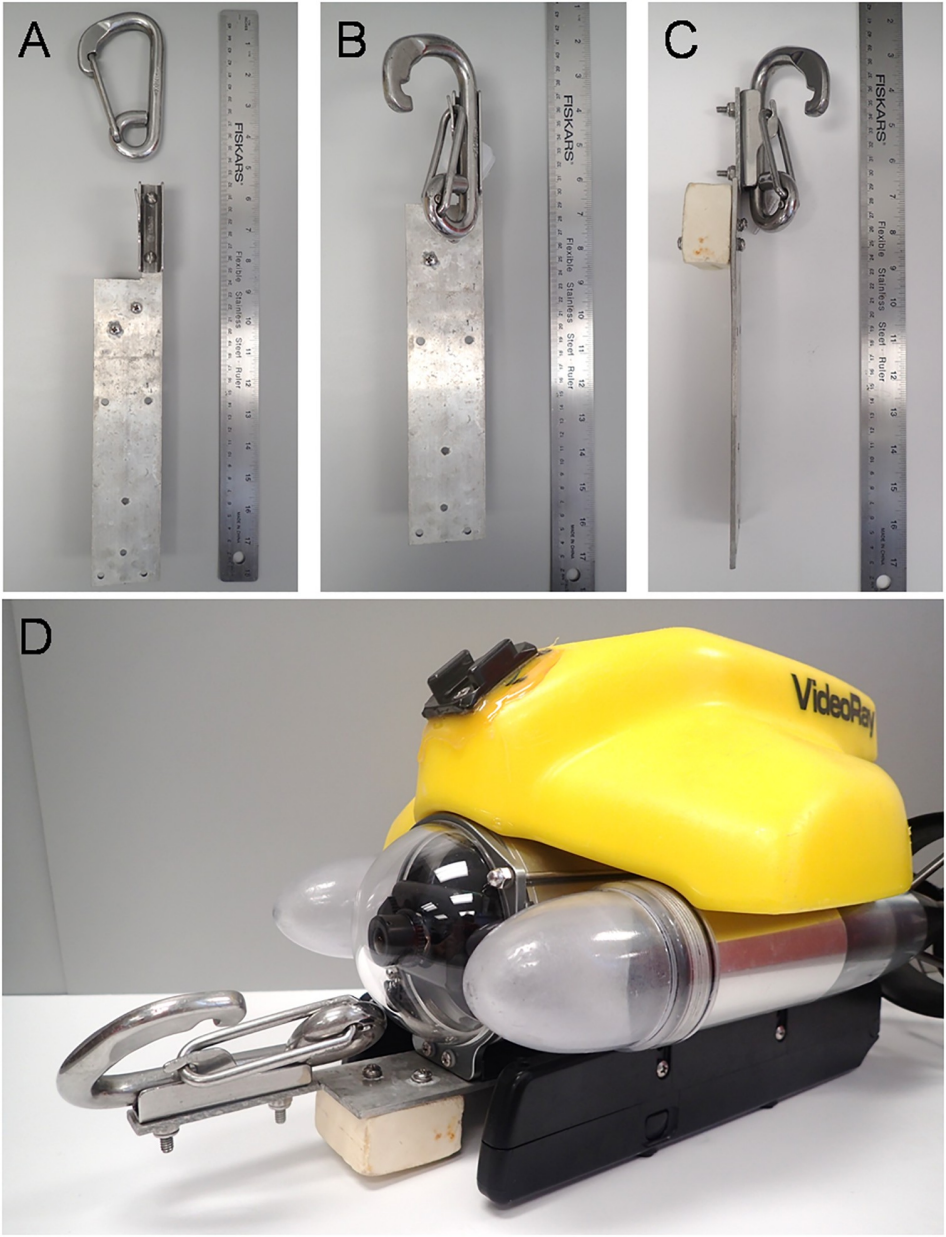
Mooring hook apparatus. A) Top view of the disassembled mooring hook apparatus that consists of a 120-mm stainless steel mooring hook and cradle (Suncor, Inc., patent US00D391474S), a 0.3-cm thick aluminum mounting plate, and syntactic foam block for buoyancy. B) Top view of assembled mooring hook apparatus. C) Side view of assembled mooring hook apparatus. D) Assembled mooring hook apparatus with aluminum mounting plate attached to the skid plate of a VideoRay Pro4 remotely operated vehicle (ROV) with the mooring hook and cradle extending in front of the ROV.

The mooring hook apparatus consists of a 120-mm stainless steel snap hook plus cradle (Suncor, Inc., patent number US00D391474S; [11]) attached to an aluminum plate that is screwed into the ROV’s skid plate (Fig 2). The aluminum plate is 0.3 cm thick, 5.1 cm wide, and 32.5 cm long, with a 2 cm x 8 cm extension of the plate along its right front edge (Fig 2a). The mooring hook cradle is 2 cm x 7.5 cm and is attached to the aluminum plate’s forward extension with two #8–32 stainless steel screws secured with nylon insert lock nuts. A 5.6 cm x 5.6 cm x 2.7 cm (256 g) block of syntactic foam with a depth rating of 750 m is attached with screws and lock nuts to the underside of the aluminum plate (Fig 2b), which provides 2.74 N of lift needed to achieve neutral buoyancy. The mooring hook cradle was altered by bending the retaining tab slightly downward. This adjustment decreased the pressure needed to pull the hook from its cradle from approximately 2.3 kg to 1.1 kg. The hook itself has a spring-loaded mechanism that closes when the hook becomes detached from the cradle. A 3-stranded nylon retrieval line with a tensile strength of 1,515 kg attached to the posterior of the hook is utilized to winch the instrument or equipment to the surface.

### Methodology

**Fig 3:**
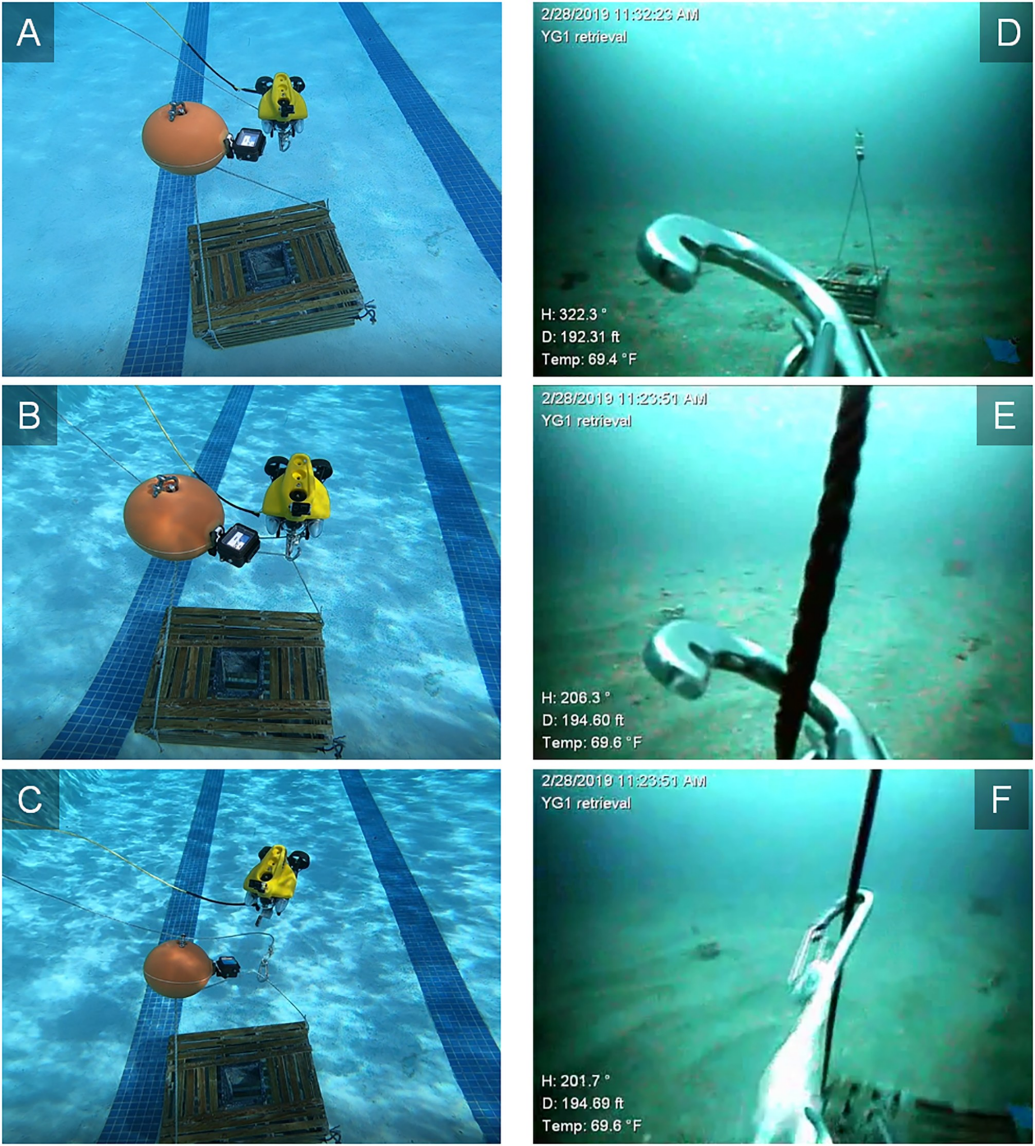
Retrieval technique. A-C) Digital images of a VideoRay Pro4 remotely operated vehicle (ROV) approaching a trap submerged in a University of Florida pool and then snatching one of the trap’s buoy lines with a mooring hook, which then released from its cradle as the ROV was flown in reverse away from the trap. Images D-F) display the same process from the perspective of the ROV’s forward camera as the mooring hook was attached to a buoy line during the process of trap retrieval from the northern Gulf of Mexico continental shelf.

The mooring hook apparatus was utilized to retrieve instruments or equipment from the nGOM continental shelf. During retrieval trips, each item to be retrieved was located by piloting the retrieval vessel to the deployment coordinates and then locating the buoy suspended 2m above it with the vessel’s sonar bottom machine. The captain would maintain the vessel’s position over the item to be retrieved and signaled to the ROV crew, which consisted of a pilot and a tether tender, to deploy the ROV. The ROV was placed in the water by hand and then flown to the seafloor. Its tether as well as the retrieval line attached to the mooring hook were paid out by the tender. The ROV pilot located the item to be retrieved by sight via the ROV’s camera once the ROV was near the seafloor. The ROV was flown toward the item and the mooring hook snatched the retrieval line attached to the item (Fig 3). The ROV was then flown in reverse and the hook was released from its cradle and snapped shut. The ROV was then flown clear of the retrieval line and the item was winched to the surface and brought on board the vessel.

## Results and discussion

**Fig 4:**
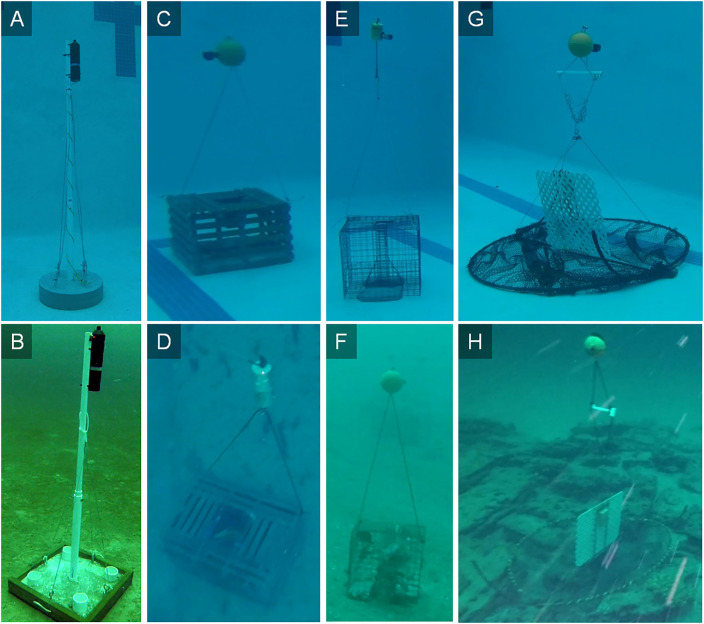
Acoustic telemetry bases and fish traps. Digital images of A&B) an acoustic telemetry base with receiver and C-H) experimental lionfish traps observed in a University of Florida pool or on the northern Gulf of Mexico continental shelf. Nylon lines (450-kg lifting strength) attached to bases or between traps and floats were snatched with the mooring hook retrieval apparatus described in the text and winched to the surface via the retrieval line attached to the mooring hook.

The ROV-integrated mooring hook apparatus and procedure described above were utilized to retrieve acoustic telemetry receiver bases and experimental invasive lionfish, *Pterois volitans*/*miles*, traps from the nGOM continental shelf. Receiver bases were constructed of poly vinyl chloride (PVC) pipes embedded in 40 kg of concrete (Fig 4a and 4e). A 2-m PVC pole was placed inside the PVC pipe embedded in the base and secured with a stainless steel bolt and nut. Four stainless steel eye hooks also were embedded in the concrete and two nylon ropes (450-kg lifting strength) were each tied to an eye bolt, extended 1.5 m up the PVC pole where they were secured with 2 50-kg cable ties, and then tied to an eye bolt on the opposite side of the pole. Acoustic receivers were attached with cable ties to the top of PVC poles (Fig 4a). A rope was also affixed to the top of poles that extended 2 m above the receiver where it was attached to a polystyrene buoy.

**Fig 5:**
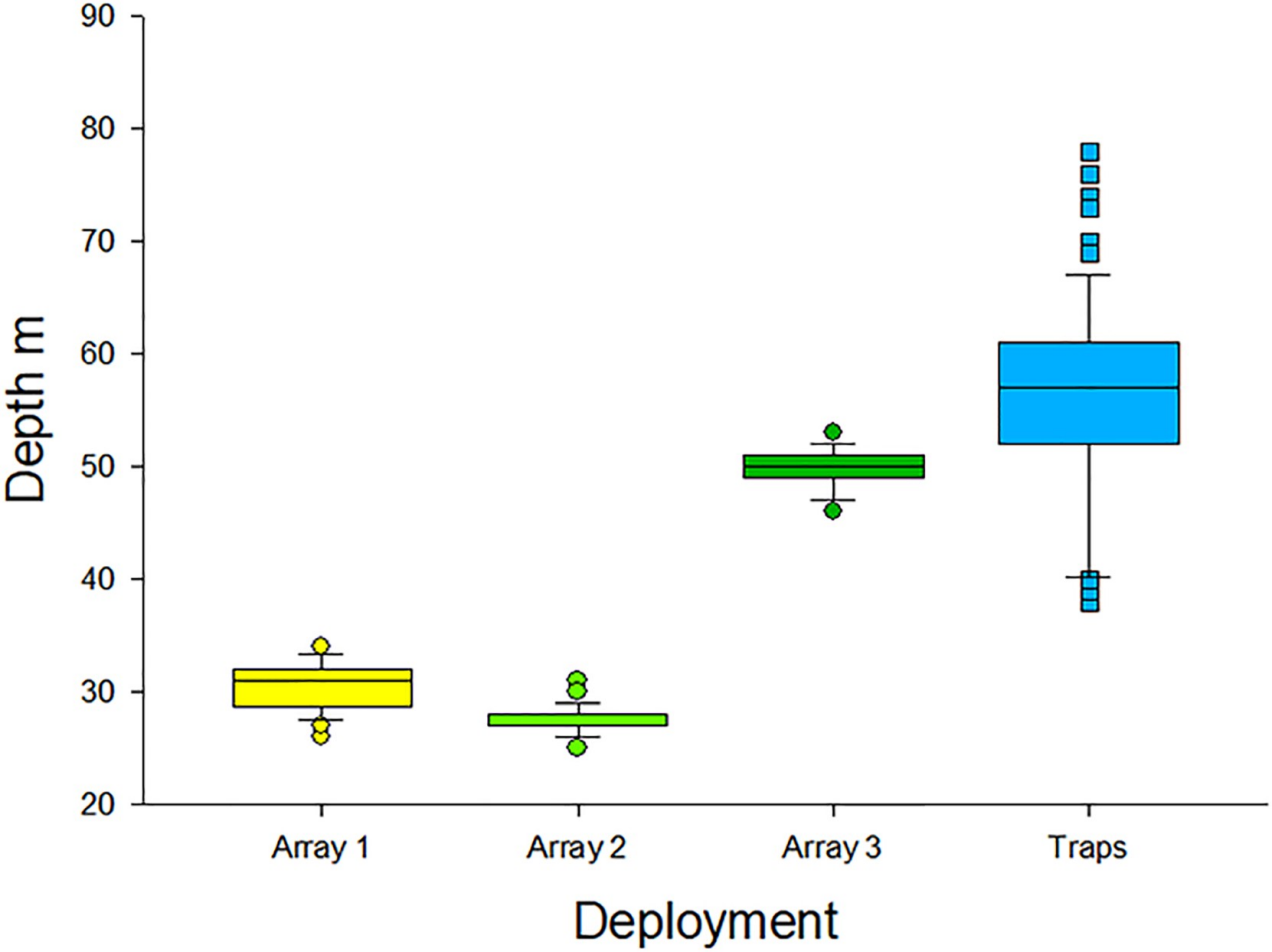
Summary of telemetry base and fish trap deployments. Box plots of depth distributions for acoustic receiver array (n = 3) deployments (n = 25, 120, and 112 individual receiver base deployments, respectively) and experimental lionfish trapping (n = 300 trap sets) in the northern Gulf of Mexico. The horizontal line inside each box indicates the median, while lower and upper sides indicate the 25^th^ and 75^th^ percentiles and extended bars indicate 5^th^ and 95^th^ percentiles. Symbols indicate acoustic receiver base deployments or traps sets that were beyond the 5^th^ or 95^th^ percentiles of the respective depth distributions.

Acoustic receivers and bases were deployed during 2013–2019 among three different acoustic arrays (n = 25, 47, and 60 receivers per array) that ranged in size between 10 and 25 km^2^ on the nGOM shelf. The depth at which receivers were deployed ranged from 28 to 64 m among arrays (Fig 5). The first array was deployed for 6 months and only involved a single retrieval. The following two arrays were deployed for 12 months each. All receivers within those arrays were first retrieved after six months, data were offloaded, and then receivers were cleaned and redeployed. Receivers were retrieved a second time at the end of each 12-month deployment. Therefore, there was a total of 239 acoustic receiver base deployments and retrievals among the three acoustic arrays.

Experimental invasive lionfish traps consisted of three different designs (Fig 4b–4d). Traps of each design had a mass of approximately 30 kg when empty but contained up to 20 kg of fish or invertebrates when retrieved. Each trap had two nylon ropes (450-kg lifting strength) that extended 2 m above it where they were attached to a buoy made of syntactic foam or polystyrene. In total, there were 300 individual trap sets (10 deployments of 30 traps each) during February-June 2019 in an approximately 1.4 x 10^3^ km^2^ area on the nGOM shelf. The depth range of trap sets was 38–80 m (Fig 4).

All of the acoustic receiver bases and experimental lionfish traps were successfully found and retrieved from the seafloor. The time between deploying the ROV and bringing a base or trap onboard the vessel was typically <15 minutes. For the smallest (first) acoustic telemetry array, all the receiver bases could be retrieved from the seafloor in a single field day. This included the running time to and from port, with a one-way distance of 32 km and a vessel speed of 22 km per hour. It took two field days, including spending one night offshore, to retrieve the two larger arrays, as well as for retrieving a single deployment of 30 lionfish traps. One-way distances to or from port for trap retrieval ranged from 30 to 75 km with vessel speeds of 22 to 28 km per hour.

The Initial cost for the ROV utilized in this work was approximately US $120,000, and ROV maintenance costs over the course of the projects described were an additional US $23,000. Those are substantial sums of money, but we estimate the total cost of utilizing the ROV-based mooring hook apparatus retrieval method described here was approximately 1/3 of the cost had we utilized divers to retrieve acoustic receiver bases and experimental lionfish traps. Furthermore, experimental lionfish traps were deployed on mesophotic (38–80 m) reefs and it would have required technical divers diving on mixed gas or with rebreathers to retrieve them. Obviously, safety issues increase dramatically when diving to those depths, and administrators at many universities or marine labs are unwilling to assume the liability of technical diving.

## Conclusions

Deploying an instrument with a surface buoy allows it to be easily located and provides a means to retrieve it, if the attached line can lift the mass of the instrument and withstand any forces required to lift the instrument from the seafloor. However, surface buoys also enable others to readily locate instruments. They can also be navigational hazards or inadvertently injure or kill marine megafauna, such as marine mammals or turtles [12]. To avoid these issues, numerous technologically extensive and intensive methodologies have been proposed to retrieve instruments from the seafloor, including everything from dragging a hook across the seafloor to snatch a cable connecting instruments and then winching them to the surface [13] to mechanical devices built specifically to lower instruments to or retrieve them from the seafloor [14]. However, acoustic telemetry and trapping studies we conducted were focused on reef fishes, hence located on or near reefs. To avoid damaging reefs or losing a retrieval device in reef structure, a more precise method was required in the cases of retrieving acoustic receivers or experimental traps from the seafloor. The methodology we describe provides such an approach while offering an alternative to long-term surface mooring or using SCUBA divers to perform frequent and dangerous dives to retrieve scientific equipment from depth. The 100% success rate on 539 deployments of traps or acoustic receiver bases in the nGOM should provide some measure of confidence that the retrieval methods described herein could be successful applied to a range of instruments or equipment deployed on the seafloor, with limitations being the mass of a given item to be retrieved, the capabilities of the ROV utilized to carry to transport the retrieval line and hook to depth, and the strength of the winch to pull the instrument to the surface.

## Supporting information

S1 File Pool experiment. Diver and ROV field of view showing the ROV’s mooring hook attaching onto the mooring line of a Gittings trap located at in the swimming pool at University of Florida.
(WMV)

S2 File Gittings trap retrieval. Successful mooring of a Gittings trap as seen from the ROV’s internal camera.
(WMV)

S3 File Lobster trap retrieval. Successful mooring of a Lobster trap as seen from the ROV’s internal camera.
(WMV)

S4 File Seabass trap retrieval. Successful mooring of a Seabass trap as seen from the ROV’s internal camera.
(WMV)

## References

[1] Clements S, Jepsen D, Karnowski M. Optimization of an acoustic telemetry array for detecting transmitter-implanted fish. N Am J Fish Manage. 2005; 25: 429–436. 10.1544/M03-224.1

[2] Butler CB, Matthews TR. Effects of ghost fishing lobster traps in the Florida Keys. ICES. J Mar Sci. 2015; 72: 85–98. 10.1093/icesjms/fsu238

[3] Schweitzer CC, Lipcius RN, Stevens BG. Impacts of multi-trap line on benthic habitat containing emergent epifauna within the Mid-Atlantic Bight. ICES J Mar Sci. 2018; 75: 2202–2212. 10.1093/icesjms/fsy109

[4] Sayer MDJ. Scientific diving: a bibliographic analysis of underwater research supported by SCUBA diving 1995–2006. Int J Soc Underw Technol. 2007; 27: 75–94. Available from: https://www.ingentaconnect.com/content/sut/unwt/2007/00000027/00000003/art00002

[5] Agar JJ, Waters JR, Valdés-Pizzini M, Shivlani M, Murray T, Kirkley JE, et al Caribbean fish trap fishery socioeconomic study. Bull Mar Sci. 2008; 82: 315–331. Available from: https://scholarworks.wm.edu/vimsarticles/1506

[6] Uhrin AV, Matthews TR, Lewis C. Lobster trap debris in the Florida Keys national marine sanctuary: distribution, abundance, density and patterns of accumulation. Mar. Coast Fish. 2014; 6: 20–32. 10.1080/19425120.2013.852638

[7] Lang MA. Scientific diving in the United States: the value of SCUBA as research methodology. Int J Soc Underw Technol. 2007; 27: 95–107. 10.3723/175605407783360044

[8] Pendergast DR, Lundgren CE. The underwater environment: cardiopulmonary, thermal, and energetic demands. J Appl Physiol. 2009; 106: 276–283. 10.1152/japplphysiol.90984.2008 19036887

[9] Sherman C, Appeldoorn R, Ballantine D, Bejarano I, Carlo M, Kesling D, et al. Exploring the mesophotic zone: Diving operations and scientific highlights of three research cruises (2010–2012) across Puerto Rico and U.S. Virgin Islands. Proc AAUS symp. 2013; 297–312. Available from: https://archive.epa.gov/region10/diving/web/pdf/2013_aaus_esdp_diving_for_science-2.pdf

[10] McMichael GA, Eppard MB, Carlson TJ, Carter JA, Ebberts BD, Brown RS, et al The juvenile salmon acoustic telemetry system: a new tool. Fisheries. 2010; 35: 9–22. 10.1577/1548-8446-35.1.9

[11] Streibel R. Snap hook. U.S. Patent No. Des. 391,474, 1998 Washington, DC: U.S. Patent and Trademark Office.

[12] Kraus SD, Brown MW, Caswell H, Clark CW, Fujiwara M, Hamilton PK, et al NorthAtlantic right whales in crisis. Science. 2005; 309: 561–562. 10.1126/science.1111200 16040692

[13] Luc F, Dowle R. Method for deploying seafloor equipment. U.S. Patent No. US 7,104,728 B2, 2006 Washington, DC: U.S. Patent and Trademark Office.

[14] Nicholls JH. Underwater equipment recovery. U.S. Patent No. 2010/0239406 A1, 2010 Washington, DC: U.S. Patent and Trademark Office.

